# No differences in histopathological degenerative changes found in acute, trauma-related rotator cuff tears compared with chronic, nontraumatic tears

**DOI:** 10.1007/s00167-022-06884-w

**Published:** 2022-02-08

**Authors:** Knut E. Aagaard, Hanna Cecilia Björnsson Hallgren, Karl Lunsjö, Richard Frobell

**Affiliations:** 1 Department of Orthopaedics, Helsingborg Hospital, Region Skåne, Sweden; 2 Orthopedics, Clinical Sciences Lund, Lund University, Lund, Sweden; 3 Department of Orthopaedics in Linköping and Department of Biomedical and Clinical Sciences, Linköping University, S-58185 Linköping, Sweden

**Keywords:** Shoulder, Rotator cuff, Acute rotator cuff tear, Trauma-related rotator cuff tear, Histopathology, Tendon degeneration, Arthroscopy, Supraspinatus tendon, Apoptosis

## Abstract

**Purpose:**

Acute trauma-related rotator cuff tears are believed to have better healing potential than chronic tears due to less degenerative changes of the tendons. However, the histopathological condition of tendons from trauma-related tears is not well investigated. The purpose of this study was to explore specific histopathological features in tendons from acute trauma-related full-thickness rotator cuff tears and to compare them to findings in tendons from nontraumatic, chronic tears.

**Methods:**

In a prospective cohort study, 62 previously asymptomatic patients [14 women, median age 61 years (range 42–75)] with trauma-related full-thickness rotator cuff tears were consecutively included. Arthroscopic repair was performed within 30 (median, IQR 25–37) days after the injury. During surgery, tissue biopsies were harvested from the supraspinatus tendons in 53 (86%) of the patients. In addition, similar biopsies were harvested from 10 patients undergoing surgery for chronic tears without history of trauma. All tissue samples were examined by a well-experienced pathologist under light microscope. Tendon degeneration was determined using the Bonar score whereas immunostaining was used for proliferation (Ki67), inflammation (CD45), apoptosis (p53) and haemosiderin staining to study traces of bleeding.

**Results:**

The median (IQR) Bonar score for the acute trauma-related biopsies was 10.5 (7.5–14.5) compared to 11 (5–12.8) for the control group with no statistically significant difference between the groups. No statistically significant between-group difference was found for the inflammatory index whereas tendons from patients with trauma-related full-thickness rotator cuff tears had statistically significantly higher apoptosis [3.1 (0.5–8.9) vs. 0.1 (0–1.5), *p *= 0.003] and proliferation [4.0 (1.8–6.9) vs. 0.4 (0–2.0), *p *= 0.001) indices than those undergoing surgery for chronic tears. Positive haemosiderin staining was found in 34% of tissue samples from patients with trauma-related tears compared to 10% in the control group (n.s).

**Conclusion:**

This study suggests that there is no difference with regard to degenerative changes between supraspinatus tendons harvested from patients with acute, trauma-related rotator cuff tears and patients with nontraumatic, chronic tears.

**Level of evidence:**

II.

**Supplementary Information:**

The online version contains supplementary material available at 10.1007/s00167-022-06884-w.

## Introduction

Pain and shoulder dysfunction are common in the general population where rotator cuff tendinopathy and rotator cuff tears (RCT) represent the most common cause of these conditions [36, 43]. A clear pathogenesis is yet to be determined but, a combination of intrinsic and extrinsic factors, gradually leading to weakening of the connective tissue leaving the tendon prone to tearing, is agreed upon [46]. Kannus and Józsa suggested already 1991 that tendon rupture is closely associated with preceding histopathological changes [19] which is supported by later studies reporting high prevalence of asymptomatic supraspinatus tears [24, 31, 33, 40, 48]. Despite this suggested overall degenerative genesis of rotator cuff tears, trauma-related tears are often studied and discussed as acute injuries and separated from chronic, nontraumatic tears [13, 29, 34, 38, 42, 50]. In trauma-related tears, early surgical repair is often advocated with the argument of superior healing properties and the advancing muscle atrophy and fatty infiltration [9, 13, 34, 43].

Although diagnostic methods and surgical techniques have developed significantly over the last decade, non-healing and retear rates of surgically repaired tendons are still unacceptably high [6, 15, 16, 35, 37]. This has led to a growing interest in aetiology of tendon tearing and biology of healing. Tendon degeneration may be estimated using magnetic resonance imaging (MRI) and ultrasound as proxies, however, histopathology of harvested biopsies from torn tendons is gold standard in determining tendon degeneration. Still, results from such trials are scarce. After a trauma-related tear, tendons are suggested to respond with a three-stage tissue healing process including an initial inflammatory phase followed by proliferative and remodelling phases [17]. Rotator cuff tendons are, however, intra-synovial and it is not known if intra-synovial tendons undergo similar healing processes as extra-synovial tendons. Nevertheless, tissue damage will most likely induce an inflammatory reaction, yet the optimal degree of inflammatory and proliferative response to facilitate healing is unknown. Apoptosis is a physiological process in healthy tissue of importance to maintain proper homeostasis. However, the role and extent of apoptosis in tissue degeneration and cuff tendon tearing is not clear and has to our knowledge not been specifically studied in trauma-related tears [3, 26, 32, 44, 49]. Further, we hypothesised that traumatic tears would show signs of bleeding that could be detected by haemosiderin staining.

Hence, the primary purpose of this study was to determine the degree of tendon degeneration in trauma-related tears with acute symptoms using the Bonar score and to compare these findings to tendons from a matched controlled group encompassing only nontraumatic, chronic tears. Secondary purposes were to study and compare the rate of inflammation, proliferation, apoptosis, and haemosiderin staining. Increased knowledge about histopathological changes and possible pre-existing tendon weakening found in previously shoulder healthy patients suffering from acute, trauma-related tears would improve the process to provide the best individual treatment.

## Materials and methods

### Patients’ selection and study design

This study involved patients included in a prospective shoulder cohort study conducted at Helsingborg Hospital in southern Sweden [1]. The study was performed in accordance with the ethical standards of the Helsinki Declaration and approved by the regional ethical review board in Lund, Sweden, registration number DNR 2011/119, 2015/36, 2016/796. Previously subjective shoulder healthy patients, aged 18–75 years, presenting at the orthopaedic or emergency department with shoulder pain, limited rotator cuff-specific shoulder function and normal plain radiographs following a direct or an indirect trauma to their shoulders formed the trauma-related group and were included in this trial between November 2010 and March 2014 [1]. Exclusion criteria were: preinjury shoulder complaints, rheumatoid arthritis, severe comorbidity or previous surgery to the affected shoulder. Diagnostic MRI was conducted within 19 (median, IQR 15–24) days after the traumatic event using the same 1.5 T scanner (Siemens Medical Systems, Erlangen, Germany) with a dedicated shoulder array coil and without contrast enhancement (Appendix I). A full-thickness RCT was defined as a discontinuity in the tendon or increased signal on T2-weighted images extending from the articular to the bursal side of the tendon [18]. All included patients were offered, and underwent, arthroscopic repair. In one of the cases, a partial repair was performed.

Surgery was performed at a median of 30 (IQR 25–37) days after the shoulder injury. All surgical procedures were carried out at the Department of Orthopaedics at Helsingborg Hospital and were performed by one of three well-experienced shoulder surgeons. During surgery, and before any radio frequency instruments was used, a biopsy of the most lateral part of the torn supraspinatus tendon was harvested in a standardised fashion using a meniscus upbiter basket punch. The tissue material was then fixed in neutral buffered formalin, transported to the Department of Pathology at Lund University where it was dehydrated and paraffin embedded. Sections of 2 μm were obtained and stained with haematoxylin–eosin (HE) and Alcian blue (AB). Sections were then scanned by Nano Zoomer S360 (Hamamatsu, Japan) and evaluated for tendon degeneration. Out of the 62 included patients that underwent surgery, 6 had intact supraspinatus tendons (isolated subscapularis tendon tears) and in 3 cases, the surgeon neglected to harvest a supraspinatus tendon biopsy leaving 53 samples for analysis. In addition, similar tissue samples were harvested from ten age- and sex-matched individuals with no history of shoulder trauma undergoing surgery for chronic rotator cuff tears. These samples formed the control group referred to as the chronic tears in this report (Table 1).

**Table 1:** Patient demographics

	Study group *n* = 53	Control group *n* = 10
Age, years (median, range)	61 (43–75)	64.5 (41–75)
Male sex (%)	81	70
Diabetes (*n*, %)	4 (8)	1 (10)
BMI (median, IQR)	28 (25–30)	29 (26–30)
Smoking (n, %)	6 (11)	3 (30)

### Tendon degeneration as determined by histopathology: the Bonar score

The Bonar score was used to evaluate the extent of tendon degeneration [10]. This score comprises a semi-quantitative grading scale that emphasises seven pathological qualities in tendinosis: fibroblastic alterations (hyper-/hypocellularity), increased glycosaminoglycan content, collagen disorganisation or disarray, hypervascularity or vascular remodelling, cell morphology, calcification, and intratendinous adipocytes. HE stains of 2-μm sections were obtained for assessment of the morphology. AB stains were performed for identification of sulfated glycosaminoglycans (GAG). The first five qualities were graded from 0 to 3 (normal to worst pathological appearance), and the presence of calcification and adipocytes are each given 2.5 points. A completely normal tendon would score 0 and accordingly, a maximally degenerated tendon would score 20 (3 × 5 + 2.5 × 2, Appendix II). The latest modified Bonar score with previously reported good inter-observer reliability (*r*^2^ = 0.71) was used [10]. All tissue samples were analysed by an experienced pathologist at the Department of Pathology, Lund University.

### Immunohistochemistry

The immunohistochemical tests were conducted at the Department of Pathology, Lund University. To evaluate the degree of inflammatory response, the expression of the pan-leucocyte marker CD45 was studied [8]. The pathologist manually marked out the borders of the entire section where counting of stain positive cells was conducted by a computer-based software (Sectra IDS7 Px, Sectra, Sweden) [21]. The inflammatory index was defined as the number of all CD45-positive cells per mm^2^.

Tendon proliferation response and activity was estimated by calculating the proliferation index [27], defined as the percentage of all Ki-67-positive cells within all fields of a given region of interest (ROI) that showed positive labelling. The pathologist visually inspected the entire section and localised a “hotspot” (i.e. the area of maximum staining) which defined the ROI. Within this ROI, counting of cells was conducted by a computer-based software (Sectra) [21].

For the assessment of apoptosis, the expression of p53 was studied. The apoptotic (p53) index was calculated and defined as the percentage of all positive p53 cells within all fields of a given tissue sample that showed positive labelling (number of positive cells/total number of all cells × 100). Here, the hotspot technique (described above) was used by the pathologist for defining the ROI.

To detect haemosiderin deposition, histochemical staining with Perls’ Prussian blue was used. No intensity scores were used; thus, any detected deposits within all fields of the tissue sample were considered positive.

Intra-observer reliability testing using intraclass correlation coefficient showed good to excellent agreement for Bonar score (ICC = 0.97), p53 (ICC = 0.96), Ki67 (ICC = 0.99), and CD45 (ICC = 0.99) as shown in Table 2.

**Table 2:** Intra-observer reliability testing ICC

	Intraclass correlation	95% Confidence interval	*p* value
Bonar score	0.97	0.89–0.99	< 0.001
p53	0.96	0.85–0.99	< 0.001
Ki67	0.99	0.99–1.00	< 0.001
CD45	0.99	0.95–1.00	< 0.001

### Statistical analysis

More than 50% of the variables were not normal distributed, and thus, median and interquartile range (IQR) is reported for all variables and nonparametric tests were used for statistical analyses. The Mann–Whitney *U* test was used for between-group comparisons, and linear regression analyses were used to study the relationship between degeneration and immunohistochemistry outcomes in the study group. Fisher exact test was used to compare the frequencies of positive haemosiderin labelling between the two groups. Intra-observer reliability was tested using the intraclass coefficient (ICC) calculation with two-way random-effects model. As suggested, an ICC below 0.50 was considered as poor reliability, between 0.50 and 0.75 moderate, between 0.75 and 0.90 good, and above 0.90 excellent reliability [22]. Significance level of 5% was considered to determine statistical significance. Statistical analyses were performed using SPSS version 24 (IBM Corp., Armonk, New York). A power calculation for the trial was done based on an 80% proportion of successful healing of repaired cuffs (regardless of number of involved tendons, primary outcome) was performed and reported [1].

## Results

**Fig. 1:**
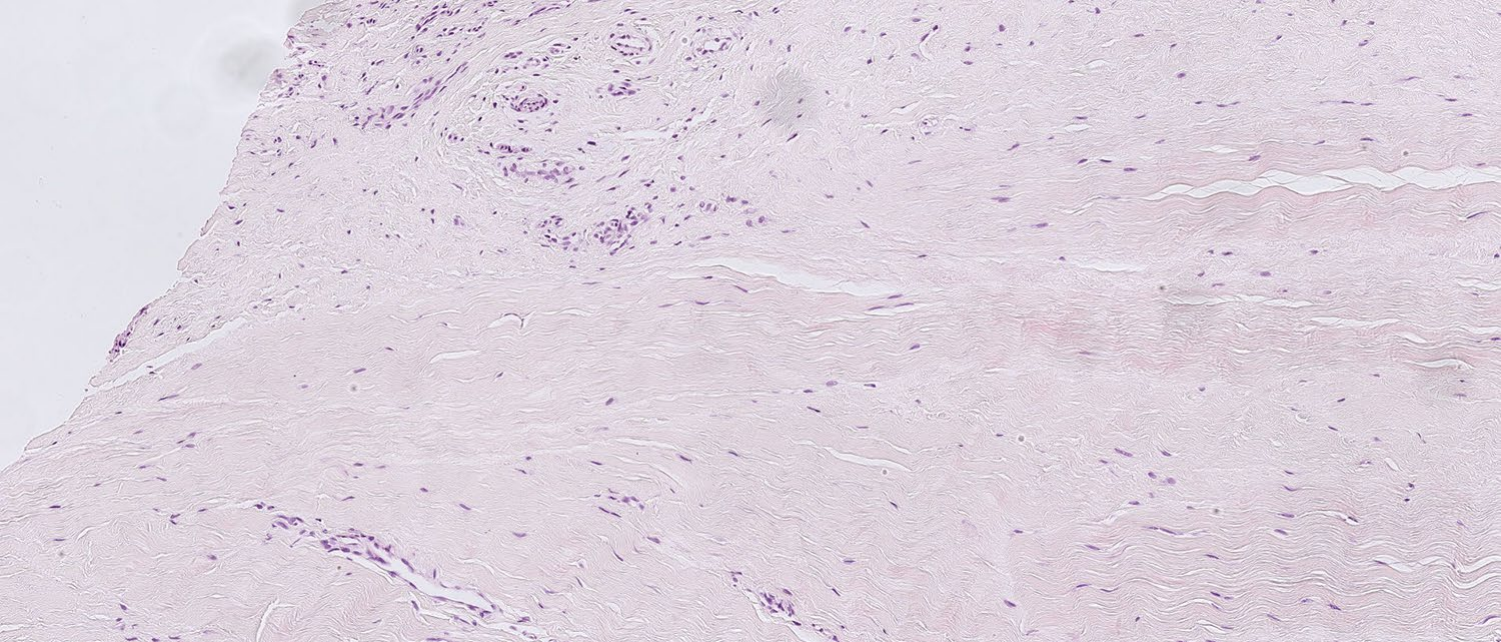
Hematoxylin and eosin stain of supraspinatus tendon harvested from the lateral edge of the tear stump in a 59-year-old woman (original magnification: × 400). Cell morphology: increased roundness, 1; collagen alignment: separation of individual fibre bundles, 1; cellularity: hypercellularity, 1; vascularity: areas of several clusters of vessels, 3; ground substance: moderate stainable GAG (graded on Alcian blue stain), 2; no calcification, 0; adipocytes detected, 2.5. Total Bonar score 10.5

The median Bonar score for patients with trauma-related tears (Fig. 1) and patients with chronic tears was 10.5 (IQR 7.5–14.5) and 11 (IQR 5–12.8), respectively, with no statistically significant difference between the groups (Table 3). There were large variations with regard to the inflammatory index in both groups, and no statistically significant between-group difference was found [2.3 (IQR 0.1–10.1) vs. 4.5 (IQR, 0.1–13.6)]. The proliferation and the apoptotic (p53) indices were statistically significantly higher in patients with trauma-related tears compared to patients with chronic tears (*p* = 0.001 and *p* = 0.003, respectively, Table 3). In the study group with trauma-related tears, the linear regression model showed a statistically significant relation between higher Bonar score and higher inflammatory index (*B* = 0.11, 95% CI [0.06, 0.16], *p* < 0.001) and higher apoptotic (p53) index (*B* = 0.13, 95% CI [0.01, 0.25], *p* = 0.04). A positive labelling for haemosiderin was found in 18 of the 53 (34%) tissue samples of patients with trauma-related tears compared to 1 of 10 (10%) in patients with chronic tears (n.s).

**Table 3:** Histopathological findings in study group compared to the control group (median, IQR)

	Study group *n* = 53	Control group *n* = 10
Bonar score	10.5 (7.5–14.5)	11 (5–12.8)
Cell morphology	2 (1–3)	2 (1–2)
Collagen arrangement	2 (1–3)	2 (1–2)
Cellularity	2 (1–2)	1.5 (1–2)
Vascularity	1 (0–3)	0.5 (0–2.3)
Ground substance	2 (2–2)	2 (1.8–2)
Calcification, *n* (%)	16 (31)	4 (40)
Adipocytes*, *n* (%)	17 (33)	3 (30)
Inflammatory index	2.3 (0.1–10.1)	4.5 (0.1–13.6)
Proliferation index	4.0 (1.8–6.9)	0.4 (0–2.0)
Apoptotic index (p53)	3.1 (0.5–8.9)	0.1 (0–1.5)
Haemosiderin staining + % (*n*)	34 (18/53)	10 (1/10)

^*^Intratendinous

## Discussion

The main finding in this first report on histopathological changes found in acute, trauma-related rotator cuff tears, was a high degree of tendon tissue degeneration. Interestingly, the degree of degeneration in the torn supraspinatus of the study cohort did not statistically significantly differ from degenerative findings in a control group with nontraumatic chronic tears. The reported high degree of tendon tissue degeneration is in accordance with Codman’s theory postulating a pre-existing tendon weakness [11]. Even though trauma may elicit an RCT, our findings support that patients with acute shoulder symptoms after a traumatic event, may have had a substantial tendon degeneration despite being asymptomatic before the injury.

Tissue damage and haemorrhage initiate an inflammatory phase, the important first step of an attempted healing process [4, 14, 17]. Prerequisites of inflammation are hematoma, deposition of fibrin, cytokine, growth factors release leading to migration of fibroblasts and endothelial cells and the inflammatory phase lasts for about 1 week [2, 12]. The large variation in inflammatory index found in our study could possibly be explained by biopsies being harvested at various time points over the first 6 weeks after trauma. Still, post hoc sensitivity analyses failed to find any relations between inflammatory index and age, time from injury, tear size or BMI (data not shown). The inflammatory response needs to be activated for healing to take place [17]; however, its role is most likely diverse in different phases of the healing process and the optimal level of inflammation to facilitate healing is not known. Several studies have reported that corticosteroids downregulate the inflammatory response and that this is deleterious to healing and repair strength [20, 30, 39, 44]. In this study, however, none of the patients received pre- or post-injury corticosteroid injections. Studies on Achilles tendons have reported that prolonged inflammation has a negative impact on healing as it disturbs the following proliferation and remodelling phases [4]. In addition, severe inflammation in normal wound healing is associated with excessive scarring [47]. Consequently, a disproportionate, pre-existing inflammation with additional inflammatory response induced by surgery may influence the healing negatively and may also induce adherences or capsulitis.

During tendon healing, the transition from inflammation to proliferation is suggested to be critical although further research is needed to fully understand the process [23]. Significantly, higher proliferation was found in patients with trauma-related tears than in those with chronic tears. Patients in this study underwent surgery, including biopsy harvesting, 20–50 days after the traumatic event and it is likely that the acute, inflammatory response of the tendon tissue was replaced by the proliferative phase in some cases. Interestingly, no correlation between tendon degeneration, as determined with the Bonar score, and proliferation index was found. This indicates that other factors determine the proliferation capability and not necessarily the degree of degeneration. However, a positive correlation between more extensive tendon degeneration and higher inflammatory and apoptosis indices was found. The positive relationship between increased levels of degeneration and apoptosis was first described by Yuan et al. [49], who moreover postulated excessive apoptosis as a primary cause of tendinopathy and tearing within the supraspinatus tendon. Results from recent studies have challenged this and in a study on supraspinatus tendinosis, Scott et al. concluded that apoptosis might play a secondary role in more advanced stages of tendinopathy, such as remodelling after tissue tearing [41]. The findings of the present study are consistent with the latter. In contrast to other publications reporting increased apoptotic index in patient with higher degree of tendon degeneration [3, 26, 27, 45, 49], significantly increased apoptotic index (p53) was found in those with trauma-related tears compared to those with chronic tears. Therefore, apoptosis in trauma-related rotator cuff tears is suggested to be associated with tendon tissue injury in addition to tendon degeneration.

Acute tendon rupture may possibly initiate bleeding although tendons are poorly vascularised. A positive haemosiderin labelling was, however, only found in one-third of the biopsies from the trauma-related tears in this study. Parts of the rotator cuff is scarcely supplied with blood and most likely, a haemorrhage is not obligate in the central less vascularised critical zone [5, 7, 25], in contrast to when an evulsion-like rupture occurs leaving a bleeding, bare footprint. Still, associated bleeding with increased healing potential is one major argument to support early repair for acute, trauma-related tears. The present results rather suggest that a trauma-related rotator cuff tear in a previously symptom-free shoulder is not necessarily associated with bleeding.

The prospective design and the well-defined study group of previously asymptomatic patients with trauma-related tears and acute symptoms are major strengths of this study. Histopathological tendon findings have to our knowledge not been reported from a respective cohort. One limitation is that the Bonar score was originally developed for patellar tendinopathy. Although it has been validated for use in rotator cuff tendinopathy [28], it has to our knowledge not been used on trauma-related cuff tears. The Bonar score findings should, therefore, be interpreted with some caution. Second, there are many other inflammatory markers than pan-leucocyte marker CD45 and the use of other markers may present different outcomes with regard to inflammation. Similarly, physiological and pathological apoptosis are highly complex processes and the p53 index is only one of several available techniques. Still, we based our selection of markers on previous rotator cuff or tendon-related studies and a previously reported valid reproducibility. Third, even though all the patients in the study group were subjectively shoulder healthy before the trauma, the preinjury tendon status was not known. It cannot be excluded that some of the patients had asymptomatic tears prior to their trauma. Still, a partial repair due to severe retraction of the supraspinatus tendon was only necessary in one patient. Sensitivity analyses were conducted with and without this patient and did not affect the outcome (Supplementary material).

Previously, asymptomatic patients suffering from trauma-related rotator cuff tears need to be informed about a likely underlying tendon degeneration and the risk of non-healing despite early surgical repair. Therefore, other factors such as tear type and size, shoulder function and patient’s activity level might be more important than a trauma-related onset of symptoms when it comes to treatment decision making in this group of patients.

## Conclusion

The results of this study suggest that acute, trauma-related rotator cuff tears in previously asymptomatic patients have pre-existing tissue degeneration, and thereby likely tissue weakening, comparable to what is found in chronic, nontraumatic tears.

## Supplementary Information

Below is the link to the electronic supplementary material.

Supplementary file1 (DOCX 12 KB)

Supplementary file2 (DOCX 1435 KB)

Supplementary file3 (DOCX 2867 KB)
