# Supplementary material for: No differences in histopathological degenerative changes found in acute, trauma-related rotator cuff tears compared with chronic, nontraumatic tears
Source: Knee Surg Sports Traumatol Arthrosc. 2022 Feb 8;30(7):2521–7. doi: 10.1007/s00167-022-06884-w (PMC9206597; doi:10.1007/s00167-022-06884-w)
Supplement: Supplementary file 1 — Supplementary file1 (DOCX 12 KB) [file 167_2022_6884_MOESM1_ESM.docx]

*Magnetic Resonance Imaging*

All MRI examinations were performed on a 1.5 T scanner (Siemens Medical Systems, Erlangen, Germany). A dedicated shoulder array coil was used. The arm was placed at the side of the body with the thumb pointing upwards. The following 7 sequences, all with a slice thickness of 3–4 mm, a 16 cm field of view (FOV), and one number of excitations (NEX) were obtained: 1) oblique sagittal T2-weighted turbo spin echo (TSE) (TR/TE = 4390/80 ms, matrix 179 × 256); 2) oblique coronal T1-wighted (TR/TE = 465/14 ms, matrix 410 x 512); 3) oblique coronal short tau inversion recovery (STIR) (TR/TE = 4720/27 ms, matrix 410 x 512); 4) oblique coronal proton density-weighted TSE with fat saturation (TR/TE = 3100/13 ms, matrix 512 × 512); 5) oblique coronal proton density-weighted TSE with fat saturation (TR/TE = 2890/94 ms, matrix 512 × 512); 6) axial proton density-weighted TSE with fat saturation (TR/TE = 3530/13 ms, matrix 512 × 512); and 7) axial proton density-weighted TSE with fat saturation (TR/TE = 3530/94 ms, matrix 512 × 512).
