# Supplementary figures and images for: No differences in histopathological degenerative changes found in acute, trauma-related rotator cuff tears compared with chronic, nontraumatic tears
Source: Knee Surg Sports Traumatol Arthrosc. 2022 Feb 8;30(7):2521–7. doi: 10.1007/s00167-022-06884-w (PMC9206597; doi:10.1007/s00167-022-06884-w)

The updated Bonar score


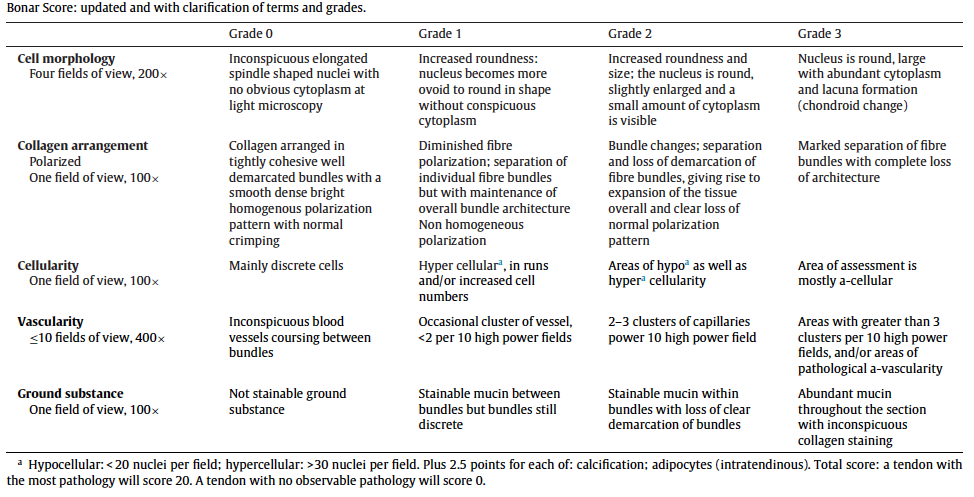

Supplement: Supplementary file 2 — Supplementary file2 (DOCX 1435 KB) [file 167_2022_6884_MOESM2_ESM.docx]
