# Supplementary material for: No differences in histopathological degenerative changes found in acute, trauma-related rotator cuff tears compared with chronic, nontraumatic tears
Source: Knee Surg Sports Traumatol Arthrosc. 2022 Feb 8;30(7):2521–7. doi: 10.1007/s00167-022-06884-w (PMC9206597; doi:10.1007/s00167-022-06884-w)

Sensitivit analyses excluding patient with partial cuff reapir (study group n=52).

Between group comparison - Bonar score vs. CD45, Ki67, and p53:


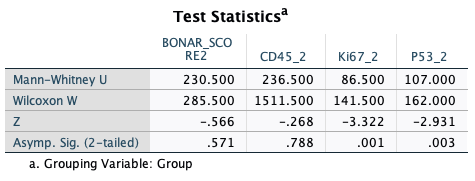


Between group comparison with Fishers´exact test for Hemosiderin :


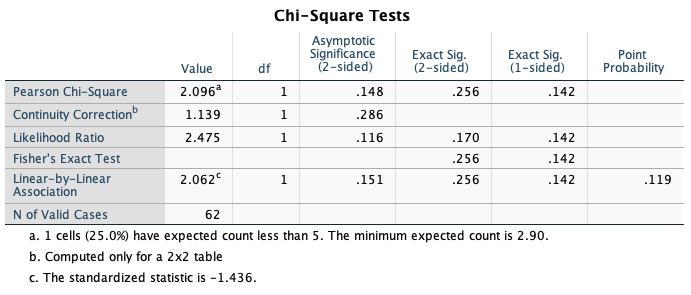


Linear regression analysis for relationship between Bonar score and CD45


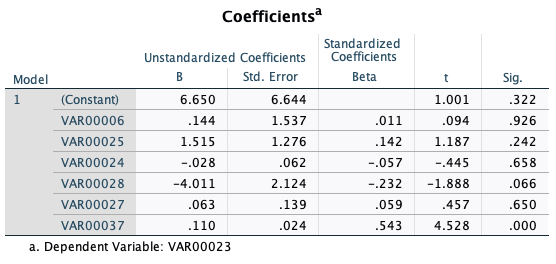


Linear regression analysis for relationship between Bonar score and Ki67


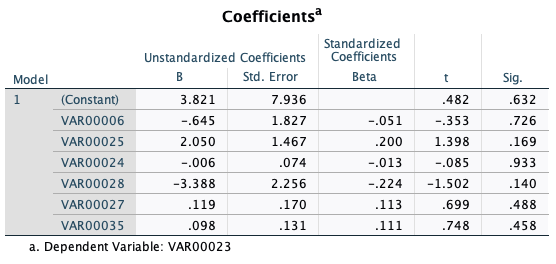


Linear regression analysis for relationship between Bonar score and p53


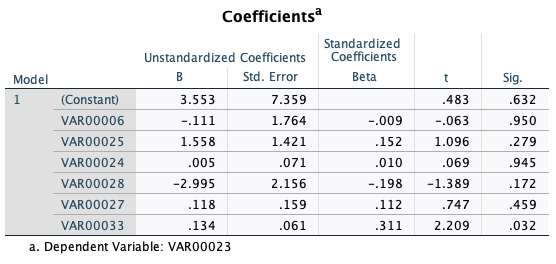

Supplement: Supplementary file 3 — Supplementary file3 (DOCX 2867 KB) [file 167_2022_6884_MOESM3_ESM.docx]
